# A dynamic architecture of life

**DOI:** 10.12688/f1000research.7315.1

**Published:** 2015-11-18

**Authors:** Beatrix P. Rubin, Jeremy Brockes, Brigitte Galliot, Ueli Grossniklaus, Daniel Lobo, Marco Mainardi, Marie Mirouze, Alain Prochiantz, Angelika Steger

**Affiliations:** 1 Collegium Helveticum, University of Zurich and ETH Zurich, Zurich, 8092, Switzerland; 2 Department of Structural and Molecular Biology, University College London, London, WC1E 6BT, UK; 3 Department of Genetics and Evolution, University of Geneva, Geneva, 1211, Switzerland; 4 Department of Plant and Microbial Biology & Zurich-Basel Plant Science Center, University of Zurich, Zurich, 8008, Switzerland; 5 Department of Biological Sciences, University of Maryland, Baltimore County, Baltimore, MD, 21250, USA; 6 CNR Neuroscience Institute, 56124 Pisa, Italy; 7 Institute of Human Physiology, Catholic University, 00168 Rome, Italy; 8 Institut de Recherche pour le Développement, UMR DIADE, Laboratoire Génome et Développement des Plantes, 66860 Perpignan, France; 9 Chaire des Processus Morphogénétiques, Centre Interdisciplinaire de Recherche en Biologie, Paris, 75231, France; 10 Institute of Theoretical Computer Science, ETH Zurich, Zurich, 8092, Switzerland

**Keywords:** computational biology, development, limb regeneration, neuroplasticity, plant epigenetics, tissue homeostasis, repair regeneration

## Abstract

In recent decades, a profound conceptual transformation has occurred comprising different areas of biological research, leading to a novel understanding of life processes as much more dynamic and changeable. Discoveries in plants and animals, as well as novel experimental approaches, have prompted the research community to reconsider established concepts and paradigms. This development was taken as an incentive to organise a workshop in May 2014 at the Academia Nazionale dei Lincei in Rome. There, experts on epigenetics, regeneration, neuroplasticity, and computational biology, using different animal and plant models, presented their insights on important aspects of a dynamic architecture of life, which comprises all organisational levels of the organism. Their work demonstrates that a dynamic nature of life persists during the entire existence of the organism and permits animals and plants not only to fine-tune their response to particular environmental demands during development, but underlies their continuous capacity to do so. Here, a synthesis of the different findings and their relevance for biological thinking is presented.

## Introduction

Life from its unicellular form to the most complex organism is increasingly understood as being composed of molecular and cellular components, which are inherently dynamic in nature and undergo specific changes in response to alterations in the internal or external environment of the organism. This insight has forced the re-evaluation of established concepts, such as cellular differentiation, as certain processes, formerly considered to occur only during development, continue life-long or can be re-induced to different degrees in the adult organism. It has also drawn anew the attention to the heritability of acquired changes and adaptations and their possible contribution to the evolution of biological systems ( Grossniklaus *et al.*, 2013; Jablonka & Lamb, 2014). From a historic perspective, one might consider the turn of the 20th to the 21st century as a period of profound conceptual transformation occurring in parallel in different fields of biological research and converging towards an increasingly dynamic conception of life ( Rubin, 2009; Sánchez Alvarado & Yamanaka, 2014).

The perception that an understanding of the basic organisation of life is emerging, which incorporates novel insights about changeability, adaptation, and heritability, has been taken as an incentive to organise an interdisciplinary workshop addressing these themes. The event took place at the Accademia dei Lincei in Rome in May 2014 and was hosted by the president of the academy, Lamberto Maffei. It is the result of a collaboration between the Collège de France, Paris and the Collegium Helveticum of the University and ETH of Zurich. The title of the event - “A dynamic architecture of life?” - was chosen to reflect the ambitions of the meeting. The juxtaposition of a wide spectrum of different scientific perspectives was sought in order to pinpoint important commonalities, but also decisive differences between the different models and empirical approaches, which are being used to investigate the dynamic nature of life and its evolution.

With the understanding in mind that it is in relation to a particular empirical context that concepts in biological research acquire a specific meaning, the aim of this workshop has not been to work towards an unambiguous and comprehensive definition of the terms in use. Instead, the interest has been in the knowledge gained about important characteristics of a dynamic architecture of life that become apparent, if the empirically substantiated articulations of the relevant concepts are presented and are discussed by experts from different fields, who usually do not interact in the framework of a workshop. The represented fields included plant genetics, evolutionary biology, developmental biology, regeneration of simple and complex organisms, the development of the vertebrate nervous system, and computational biology. The presentations on biological research were complemented by a commentary from a historian of science. The discussions produced a rich and very differentiated picture on the multiple ways the dynamic complexity of life has arisen during evolution. In reflection of the theme of the conference, one can state that all contributions to this review ascertain that an understanding of plant and animal physiology as largely static, determined by fixed set points and unchangeable structures, is no longer considered valid. In contrast, ample evidence is provided for a persisting potential of organisms to adapt to novel functional requirements life-long. This potential is tightly regulated and differentially activated by a wide range of external and internal signals and is being discussed as subject to selection during evolution. In the following, you will find short introductions to the different topics, followed by reviews of the different fields, and a general discussion.

## Epigenetics

During the past decades, DNA, which might be considered the central element of a dynamic architecture of life, has been documented as a site of both targeted and stochastic changes. These changes, as presented by **Ueli Grossniklaus** and **Marie Mirouze**, include genetic and epigenetic alterations that can also be interrelated, including the reorganization of the genome by transposable elements and a wide spectrum of epigenetic modifications, such as the methylation of cytosine in DNA or various modifications of the proteins associated with DNA. The epigenome is considered an important intermediate step between the genetic information and its expression, permitting differential interpretation of the primary sequence in different cells during development. The frequency and reversibility of epigenetic changes make them attractive candidates to mediate relatively rapid adaptation of plants to changes in their environment. However, in which ways these epigenetic changes are transmitted and influence the evolutionary trajectory of a species, be it in plants or in animals, is still a matter of debate and will require further empirical work.

## Regeneration

The capacity for regeneration is present amongst species belonging to a broad range of phyla. This widespread occurrence implies that - over evolutionary time – natural selection has favoured species capable of partial or complete regeneration in response to various circumstances. **Jeremy Brockes** presents the salamander as an instructive system, which permits to study the conditions and characteristics of limb regeneration and their correlation to particular developmental features. In respect to a dynamic architecture of life it is noteworthy that no senescent cells persist after repeated limb ablation, due to a specific mechanism of surveillance, permitting the repetitive reconstitution of a range of tissues. This relates the phenomenon of limb regeneration to the homeostatic and developmental plasticity of the fresh water polyp hydra, whose different contributions to the maintenance of the body architecture are being discussed by **Brigitte Galliot**.

## Plasticity

**Marco Mainardi** presents the regulation of energy metabolism in mice as much more dynamic than previously understood, reflecting the plasticity of the developing and the adult nervous system. His analysis underscores the interplay of physiological, social, and cognitive demands in shaping the functionality of the hypothalamus, as a key regulator of lipid metabolism. Accordingly, possible strategies to alter the plasticity of specific brain areas for therapeutic purposes are being discussed. Importantly, a critical period of heightened plasticity and sensitivity to external signals during development seems one amongst several features of a dynamic neuronal architecture common to both cortical and subcortical structures of the mammalian brain. The critical period of visual development has been very instructive in dissecting the interplay “between nature and nurture” in shaping the visual cortex. **Alain Prochiantz** points out that the homeodomain transcription factor Otx2 is both necessary and sufficient for initiating and terminating the critical period of increased malleability of the visual cortex. The possibility to modify the onset and termination of the critical period by Otx2 and other factors intrinsic and extrinsic to the nervous system reflects the fact that plasticity constitutes the tightly controlled default state of the adult cortex.

## Computational biology

The opportunities, but also the demanding tasks to realistically model the different aspects of a dynamic architecture of life based on a range of empirical data, become apparent in the contributions by **Daniel Lobo** and **Angelika Steger**. Lobo uses the possibility of reverse engineering to group and order a large quantity of existing empirical data in an encompassing model on regenerative events in the planarian flatworm. This model is automatically inferred from experiments described in an unambiguous mathematical language. It can be used to predict the results of novel interventions in the modelled organism, which can then be validated by further empirical work. Steger applies modelling to analyse information storage in the brain. Her work leads her to propose that variability of parameters representing neuronal functions has important ramifications both in terms of increased speed of signal transmission, but also heightened stability of the signalling processes within recurrent networks, as well as lowering energy demands. Such models complement data gained from empirical work by addressing the relevance of specific factors and parameters in processes of regeneration and plasticity.

## Epigenetic variation within and across generations (Ueli Grossniklaus)

Over the last decade the term “epigenetics” has appeared more and more often in the popular press and has been linked to effects on health or behaviour that have been claimed to depend on particular circumstances that our parents or grandparents had experienced. Such reports of “non-genetic” inheritance have refuelled the debate on the role of “nurture versus nature”. In this contribution it will be discussed in which ways epigenetic regulation might contribute to a dynamic architecture of life by rendering the nature of DNA and its functions more variable.

So what is epigenetics? In fact, it is an old concept in developmental biology dating back to the 1940’s that has gained great momentum over the last 15 years, as many of the underlying mechanisms have been unravelled. Originally, Waddington defined epigenetics as ‘‘the branch of biology which studies the causal interactions between genes and their products which bring the phenotype into being’’ ( Waddington, 1942; Waddington, 1968). In the era of molecular biology, the focus has shifted to the hereditary material and a commonly used definition states that epigenetics concerns the “study of mitotically and/or meiotically heritable changes in gene expression that occur without a change in DNA sequence” ( Riggs *et al.*, 1996). Thus, in principle, epigenetic regulation plays a role at two levels. First, it is involved in development – within a generation – leading to the specification of cells and assuring the faithful inheritance of their differentiated state through a series of mitotic cell divisions. Second, epigenetic states can be inherited meiotically – across generations – endowing the progeny with information gathered during the lifetime of the parents. While the former is widely accepted and contributes – due to the metastable nature of epigenetic variation – to both developmental stability and plasticity, the relevance of the latter is being debated. Most evolutionary biologists do not attribute a role to epigenetic variation in evolutionary processes. However, the more rapidly changing nature of epigenetic variation makes it particularly attractive for adaptations to a changing environment.

Despite the great attention that transgenerational epigenetic inheritance has gained in the popular press, there have been only a few – often controversially discussed – cases documented in mammals. Environmental influences that can be traced back to parents or grandparents are widespread, but they might be mediated by a wide variety of mechanisms and the underlying nature may or may not necessarily be epigenetic. Controversies arise because many authors do not distinguish between (grand)parental effects and true transgenerational inheritance, which must be demonstrated to occur in several generations after the exposure to a particular environmental condition. For instance, if a pregnant mouse is exposed to a particular treatment, e.g. a specific nutrient regime, not only the mother but also the fetus (F1 generation) and the germ line cells set aside in this foetus that will give rise to future offspring (F2 generation) will be affected by the same treatment. Thus, effects in the children and grandchildren of the treated mouse are not considered as evidence for transgenerational epigenetic inheritance and are now often referred to as “intergenerational”. In cases where effects on subsequent generations were studied in detail, the effects disappeared in the great-grandchildren (F3 generation), leading to the conclusion that they were not transgenerational in nature ( Radford *et al.*, 2014; Waterland *et al.*, 2007). Nevertheless, there are some intriguing cases of potential transgenerational effects in humans ( Pembrey, 2010) that were identified in epidemiological studies, whose underlying nature has not yet been investigated further.

In contrast to the scarce data in mammals, there are many well-studied examples of stably inherited epialleles in round worms ( Guerin *et al.*, 2014) and plants ( Hirsch *et al.*, 2012; Paszkowski & Grossniklaus, 2011). While the (meta)stable inheritance of epiallelic variants is firmly established in plants, it is not clear whether specific treatments (e.g. heat stress) cause epigenetic changes that can be passed on to the next generation and lead to offspring that is better adapted to the specific conditions the parental generation has been exposed to. While there are several publications reporting adaptation to specific biotic and abiotic stresses in the progeny of treated plants, these experiments have been difficult to reproduce and such an adaptive memory does not seem to be a general response to stress in plants ( Hirsch *et al.*, 2012; Pecinka & Mittelsten Scheid, 2012). However, in plants new epigenetic variation is generated in each generation, e.g. by spontaneous changes in cytosine methylation ( Becker *et al.*, 2011; Schmitz *et al.*, 2011), and it seems that – just like genetic variation – epigenetic variation can also be selected upon (Heichinger and Grossniklaus, unpublished). As epigenetic variation in a population is much higher than genetic variation, epialleles may indeed contribute to the rapid adaptation of plants to novel environmental conditions, provided the epigenetic change is stable enough. Indeed, there are yet unpublished observations that plants of the genus *Diplacus* can change the colour, morphology, and pollinator under certain environmental conditions and that this change is heritable over several generations (Baumberger and Grossniklaus, unpublished). Since this epigenetic switch leads to a change in pollinator, and thus establishes a reproductive barrier, it will affect the evolutionary trajectory of this species, suggesting a role of epigenetics in ecology and evolution ( Hirsch *et al.*, 2012).

Although many of the mechanisms underlying epigenetic regulation have been unravelled over the last 15 years, the role and importance of epigenetics in ecology and evolution still remains unclear. In the future, it will be interesting to see whether non-genetic information influenced by the environment indeed plays a role in adaptation and how important and wide-spread such processes are in comparison to genetic ones.

## Genome plasticity and the underestimated role of mobile genetic elements (Marie Mirouze)

The genome has long been seen as a static entity, transmitted throughout generations with high fidelity, and only rearranged in sexual organisms through the mechanisms of crossing-over occurring during meiotic recombination. The discovery made by Barbara Mc Clintock that small pieces of the genome (called Transposable Elements, TEs) could be mobilised and, upon stress, induce chromosomal rearrangements in the absence of meiotic recombination had broken the dogma in the early fifties ( McClintock, 1984). Later the extensive genome sequencing projects have revealed that TEs make up most of sequenced eukaryotic genomes, from 45% in the human to up to 85% in the maize genome, revealing that big genomes reflect a high amount of TEs and not a difference in gene content.

The studies of genome expression have revealed another layer of complexity in the last decades, as a code on top of the genomic code, hence called the epigenome (epi=above). Conrad Waddington had been the first one to coin the term epigenetics to describe how the action of genes could give rise to a landscape of different developmental possibilities that would determine the cell fate during embryo development ( Slack, 2002). Although his concept was visionary, he was not aware of the molecular details of what would become the focus of research of a large scientific community decades later. The numerous studies on epigenetic marks, chemical modifications of the DNA sequence itself, or on the histone proteins around which the DNA is wrapped, have shown that the epigenome is at the interface between the code (the genome) and its function (expression). Mammalian X chromosome inactivation ( Chaligné & Heard, 2014) or flowering time control in plants ( Song *et al.*, 2012) are famous examples in which expression of genes are prevented by epigenetic mechanisms during development. Because of their plurality and versatility these epigenetic marks can vary in different cells and tissues within one organism, hence allowing different interpretations of the genomic code during development but also upon stress. Recent studies have further contributed to demonstrate that the manipulation of the epigenome in wild-type organisms (plants in this case) leads to the release of the epigenetic silencing of TEs and to their active mobility in the genome across generations ( Mirouze *et al.*, 2009). As expected for a biological phenomenon, there is not only one, but several different and complementary mechanisms that contribute to TE silencing under normal conditions. Hence the epigenome can be seen as a guardian of the genome, preventing uncontrolled TE mobility that would affect the host fitness. Nevertheless, TEs have been successful in proliferating in most eukaryotic genomes, and their role, if not yet clear at the organismal level, has to be envisioned at the evolutionary scale. Indeed owing to their inherent ability of movement, TEs represent a molecular force in evolution. Three specific features of TEs have been shown to contribute to genome evolution. Firstly some essential functions (DNA sequence recognition through transposase, insertion in the genome through integrase) have been co-opted or domesticated by different host genomes and have led to the evolution of important molecular functions such as the telomerase in eukaryotes or the apparition of adaptive immunity (CRISPR in prokaryotes and V(D)J recombination in mammals are good examples, see Koonin & Krupovic, 2015). Secondly, as transcribed entities, TEs possess regulatory *cis* elements that can be amplified in the genome and have an impact on neighbouring genes leading to the rewiring of regulatory networks ( Feschotte, 2008). This is best exemplified in the apparition of the placental function in mammals ( Lynch *et al.*, 2011). Finally, the mobility of TEs themselves has been shown to contribute to genome plasticity in the brains of mammals ( Baillie *et al.*, 2011). The causes and consequences of the mobility of some TEs in the human brain are not yet clear. However, these studies illustrate another break in the dogma of genomic stability: multicellular organisms are not composed of one but of a myriad of slightly different genomes, the germline genome being most likely the most stable and best protected against TE mobility. Cancer cells with uncontrolled genome plasticity represent an extreme, and the role of TEs in their proliferation is just being investigated ( Lee *et al.*, 2012). In normal cells, genome plasticity might have a role in development but this is yet to be discovered. Single cell sequencing together with targeted sequencing of active TEs should allow us to better appreciate the extent of genome plasticity and the role played by TEs.

## Mechanism and evolution in vertebrate limb regeneration (Jeremy P. Brockes)

The ability to regenerate complex structures in an adult animal is indicative of an underlying dynamic architecture of life. In vertebrates, the most extensive repertoire of regeneration is found in the urodele amphibians or salamanders. This includes most notably the limbs, and salamanders are the only adult tetrapods able to regenerate these appendages. Studies on the mechanism of limb regeneration have considered aspects such as the reprogramming of differentiated cells, and the requirement for concomitant regeneration of peripheral nerves ( Brockes & Gates, 2014). These features of a dynamic architecture have been of considerable mechanistic interest, as well as providing valuable pointers for extending the possibilities of mammalian regeneration. The central, and most difficult, questions are concerned with limb regeneration as an evolutionary variable. For example, should limb regeneration be regarded as an ancestral property of tetrapods, that was lost in anurans and amniotes, or did it evolve as a new property in the salamander lineage? The answers to the evolutionary questions encompass other noteworthy issues, such as the relationship between the regeneration of the adult limb, and its embryonic and larval development. In my contribution to the workshop I considered first a rather understudied aspect of the mechanism, which provides interesting pointers for mammals, and second recent evidence on limb regeneration as a salamander novelty ( Garza-Garcia *et al.*, 2010).

One characteristic of regeneration in animals such as salamanders is the ability to undergo multiple sequential episodes with minimal change in outcome, even in some cases in ageing animals. This has recently been shown in relation to newt lens regeneration, in which a cohort of animals was followed over eighteen annual episodes ( Eguchi *et al.*, 2011). There have also been several studies of fin regeneration in the zebrafish that have explored this property and concluded that the outcome of regeneration cannot be limited by replicative senescence. The state of cell senescence was originally identified in relation to the arrest of proliferation that occurred after a certain number of divisions in culture, and it has also been implicated as an anti-tumour mechanism. More recently, senescent cells have been found in certain contexts in mouse development, for example the kidney and the limb, suggesting that we do not fully understand the significance of this state. We have established a culture system where newt limb cells are induced to enter senescence, and display a variety of markers found in mammalian senescence. This allowed us to show that a cohort of senescent cells are induced early after transection of the salamander limb, but they are removed effectively during the course of one cycle of regeneration ( Yun *et al.*, 2015). The ability to implant normal and senescent cells from culture into the limb has allowed us to analyse this process, and to determine that it is dependent on macrophage-mediated removal. Senescent cells accumulate in mammals with age, and there is some evidence that genetic ablation of senescent cells may ameliorate certain age-related disorders in mice. The salamander is apparently an animal with an active and efficient mechanism of surveillance that removes senescent cells and prevents their accumulation, even after multiple cycles of limb regeneration. Nonetheless, it is possible that the transient cohort may play an important role in regeneration. We think that this context provides a valuable perspective for efforts to target senescent cells in mammals, as well as underlining a new element of the dynamic architecture of the limb that supports regeneration.

If limb regeneration is an ancestral property in vertebrates, it might have persisted in salamanders from fin regeneration following the fin-limb transition. It would then have been lost in anurans and amniotes. Alternatively, the regeneration of the limb may have presented a barrier that required additional evolutionary novelty over the background level of regeneration present in salamanders. Note that both views of limb regeneration imply selective pressure for this property in salamanders, either to maintain it, or to make the transition from a non-regenerative state. One line of evidence that supports some contribution of 'local' evolution is the identification of salamander-specific proteins in regenerating limbs. This includes the salamander orphan gene *Prod 1*, a member of the Three Finger Protein (TFP) superfamily, and previously implicated in nerve dependence and positional identity ( Brockes & Gates, 2014; Garza-Garcia *et al.*, 2010). *Prod 1* is probably present in all salamanders, as it has recently been identified in one of the basal families, the Hynobiidae, which diverged in the early Jurassic. Other putative taxon-specific genes expressed during regeneration have been identified in proteomic and transcriptomic databases from salamanders ( Looso *et al.*, 2013). While this microevolutionary evidence is a valuable inroad for our understanding of regeneration, more functional studies will be necessary to determine whether these genes are embedded in the mechanism.

There are certain established aspects of limb development in salamanders that are different from other tetrapods ( Fröbisch & Shubin, 2011). These relate to the order of appearance of the digits, and the order of their subsequent ossification. It is often pointed out that limb development occurs after hatching in many aquatic salamander larvae, and the developing limb is subject to selective pressures for anchorage, balance, and propulsion. These pressures might have extended to engendering its regeneration after loss by injury or predation, and illustrate the interaction between the organism and its environment. The precocious extension of digits 2 and 1 may be critical to allow rapid elongation of the developing limb in this context. In contrast to regeneration, there is a clear consensus that these differences in timing, referred to as preaxial dominance, represent a salamander novelty imposed on the mechanism of limb development found in other tetrapods, termed postaxial dominance. It has been suggested that there is a connection between the evolution of this property, and that of limb regeneration ( Brockes, 2015; Fröbisch & Shubin, 2011). The relevance of preaxial dominance to regeneration, and whether it occurs during regeneration as well as development, are both unclear. An intriguing recent contribution comes from the analysis of the fossil record of Paleozoic amphibians. Preaxial dominance is readily visible in the developing bones of the limbs, and is clearly detectable in fossil larvae of the temnospondyl species Apateon in the early Permian (about 300 MYA). It has recently been reported that fossils of Micromelerpeton, a close relative of Apateon and inhabiting the same mountain lakes, show evidence for limb regeneration in the presence of certain characteristic abnormalities in the limbs ( Fröbisch *et al.*, 2014). It is possible that regeneration and preaxial dominance originated in the same species at a time close to the divergence of the salamander lineage ( Brockes, 2015). Our ability to promote mammalian regeneration will be aided by further understanding of the evolution of regeneration, and the factors underlying the dynamic architecture of the salamander limb.

## Plasticity of homeostasis and regeneration: what can we learn from *Hydra*? (Brigitte Galliot)

How do we maintain the shape and functionalities of our body over years? As adult organisms, we continuously replace - in the absence of any stress or injury - tissues thanks to genetic programs that strictly control the size, the anatomical organization, and the function of these tissues. Such replacement processes that characterize the maintenance of homeostasis occur at paces specific to each organ, typically slow for brain, lungs, kidneys, and rapid for intestine, blood cells, and skin. Genetic programs are constrained cascades of events orchestrated in nuclei by transcription factors, RNA-binding proteins, microRNAs, long noncoding RNAs, which all together modulate the activity of target genes whose activities modify the behaviours of cells and tissues. An excellent example of such a genetic cascade is the *Hippo* pathway, initially identified in *Drosophila*, which similarly regulates the homeostasis of numerous organs in planarians and mammals ( Piccolo *et al.*, 2014).

In conditions of stress or injury, exogenous cues play a key role to trigger immediate responses involved in wound healing, and to activate evolutionarily conserved genetic programs that repair damaged tissues or injured organs by restoring size, anatomy, and function but also prevent degeneration and uncontrolled growth ( Cordeiro & Jacinto, 2013; Vriz *et al.*, 2014). However, these repair mechanisms reach some limits as the genetic programs we used to develop during our embryonic life progressively close to become, in most cases, irreversibly inaccessible in adulthood. This implies that repaired tissues are often not fully restored, as skin or hearts that become fibrotic after repair and poorly functional. This also implies that we are not able to reconstruct complex structures such as limbs or even complex organs as kidneys, heart, and lungs. When I say “we” I mean the large mammalian family, as most mammals including humans exhibit limited regenerative abilities. By contrast a cohort of non-mammalian species spread over a wide phylogenetic spectrum, including sponges, cnidarian polyps, planarians, annelids, crustaceans, echinoderms, urochordates, fish, salamanders, and lizards, is able to regenerate missing body parts, amputated appendages, and destroyed organs.

One possible hypothesis is that most metazoans are highly plastic as adult organisms, a feature subsequently lost in mammals. Here, I will consider two forms of plasticity, homeostatic and adult developmental, both taking place in fully developed sexually mature organisms. The term “homeostatic plasticity” refers to the adaptation of an intact organism to external or internal changes, whereas “adult developmental plasticity” refers to the possibility for adult organisms to trigger developmental programs upon injury or damage ( Galliot & Ghila, 2010). These programs usually share similarities with embryonic developmental programs, but the main difference is that they are used in a fixed and highly constrained manner during embryonic development, but with much plasticity during adulthood.

**Figure 1.**
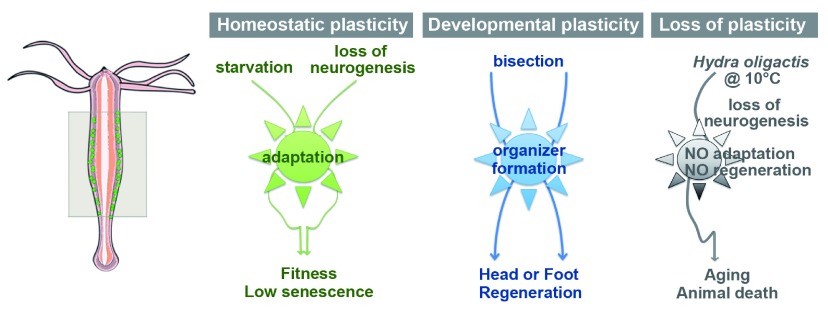
Homeostatic and developmental plasticity in *Hydra*. The scheme represents the typical epithelial bilayered anatomy of *Hydra* polyps with interstitial stem cells (green dots) distributed along the central body column (framed box). By contrast cells at the apical and basal extremities are terminally differentiated. A highly dynamic homeostasis with continuous renewal of stem cells supports the fitness and the low senescence of *Hydra*. Animals survive long periods of starvation as well as the elimination of the nervous system if force-fed. After bisection at any level along the body column, the stump is able to reestablish an organizer centre and regenerate the missing part, either basal or apical. However, some *Hydra oligactis* strains show a very low level of plasticity: they do not adapt to the loss of neuronal progenitors induced by the transfer to cold temperature, they rapidly lose the ability to regenerate, undergo aging, and finally die in a couple of months.

*Hydra* is a small freshwater cnidarian polyp that provides a unique model for investigating homeostatic and developmental plasticity: these animals remain fit over weeks of starvation, and do not display signs of senescence when regularly fed. Moreover they reproduce asexually through budding, they regenerate any missing part when bisected along the body axis, and they can even reaggregate and reshape to form the original polyp shape when dissociated as a cell suspension ( Galliot, 2012). They also survive the elimination of their nervous system becoming “epithelial” but are still able to bud and regenerate if force-fed. All this is possible due to three large pools of stem cells that continuously divide and provide differentiated cells. So *Hydra*, which can easily be propagated as mass culture, provides a model system for monitoring a highly dynamic homeostasis, the response to change in environmental conditions (feeding, temperature), the response to injury, and the reactivation of two distinct developmental programs, head and foot regeneration ( Figure 1). Interestingly, a large number of genes are conserved from *Hydra* to humans ( Wenger & Galliot, 2013) and studies over the past 25 years convincingly showed that the genetic programs active in *Hydra* often play similar functions in *Drosophila* and mammals, as for example neurogenic genes ( Galliot *et al.*, 2009; Miljkovic-Licina *et al.*, 2007).

However, some *Hydra oligactis* strains undergo a rapid aging process when transferred to cold temperature ( Tomczyk *et al.*, 2015; Yoshida *et al.*, 2006). After few weeks these animals lose (i) their highly dynamic homeostasis as evidenced by the decrease in sustained proliferation of epithelial stem cells, (ii) the ability to adapt to a stressful environment as shown by the dramatic impact of the loss of neurogenesis, and (iii) the ability to regenerate missing body parts (Tomczyk *et al.* in preparation). As a consequence, these animals lose their fitness in a month and survive no longer than two months. Interestingly, the dissection of the molecular and cellular mechanisms underlying aging in such *Hydra* points to processes highly reminiscent of those active in humans.

In summary, *Hydra* provides a potent model system to understand how a dynamic homeostasis can support a robust adaptability to environmental changes, and how developmental plasticity can promote regeneration. Each of these properties has biomedical implications of utmost interest, as the characterization of the mechanisms underlying plasticity in *Hydra* should help identify candidate regulators of regeneration and aging in humans.

## Plasticity in hypothalamic circuits controlling leptin sensitivity and food intake (Marco Mainardi)

Metabolic homeostasis has been traditionally thought to be a static process, characterized by a relatively narrow range of physiological and behavioural responses to the abundance or scarcity of nutrients, e.g, regulation of glycaemia, food seeking, and food intake. However, neurons have been shown to change their activity in response to changes in the levels of metabolic hormones ( Mainardi *et al.*, 2013; Mainardi *et al.*, 2015). In addition, the basal hypothalamus, a key area for energy homeostasis, has recently been shown to host a new neurogenetic niche ( Kokoeva *et al.*, 2005), along with the subventricular and subgranular zones (see also Prof. Prochiantz’s contribution). Thus, regulation of metabolism can definitely be considered as a plastic and dynamic process, leading to a substantial revision of the concept of a “set-point” for metabolism, towards a more dynamic view.

The plastic potential of the hypothalamus is an essential feature for the adaptation of neuronal output to variations in environmental stimuli, in close similarity with the role of sensory experience in shaping the cortex. Indeed, brain areas taking part in metabolic homeostasis have to adjust their response not only to drive food-seeking behaviour, but also to maintain a proper amount of energy stores. The net result of this process regulates the balance between energy accumulation and expenditure, thus giving an important contribution in determining body weight and metabolic activity.

Our previous work ( Mainardi *et al.*, 2010) has shown that the external environment can dynamically affect the development of hypothalamic neurons’ sensitivity to leptin, the hormone secreted by adipose tissue to negatively regulate food intake and stimulate energy expenditure ( Friedman, 2014). In these experiments, mice were exposed to environmental enrichment since birth to enhance the motor, cognitive, and sensory stimulation. Under these conditions, a reduction in circulating leptin levels was observed, which, surprisingly, did not produce the expected increase in food intake. This finding was suggestive of enhanced leptin sensitivity and, consistently, neurons located in the hypothalamus were more responsive to this hormone. In addition, food intake repression by leptin was accentuated in comparison to mice reared under standard conditions. Taken together, these results indicate that the quantity and quality of external stimuli are able to modulate the development of the set-point for lipid homeostasis.

At the synaptic level, we found rearrangements in the excitation/inhibition ratio of hypothalamic synapses, which indicated a shift towards activity patterns aimed at repressing food intake. This structural observation was put in correlation with a higher expression of the gene encoding for BDNF, a neurotrophin and a master regulator of neural plasticity.

One of the most interesting aspects is that synaptic plasticity was not observed if mice were exposed to physical exercise alone (although leptin sensitivity was enhanced at a comparable level), indicating that cognitive stimulation and social interaction also have an important role in determining the metabolic set-point.

A striking aspect is that programming of leptin sensitivity by environmental stimuli was shown to be possible only during early postnatal development, suggesting the existence of a critical period, as it has been described for sensory cortices. The analogy with the cortex also extends to the main mediators of plasticity: modulation of the excitation/inhibition ratio and BDNF abundance.

This parallel between cortical and sub-cortical areas leads to the speculation that the plasticity of functionally distinct brain districts is actually regulated according to the same general, unifying principles. According to this view, the same array of molecules are organized into a common framework that provides the substrate for generating the dynamic architecture of the brain.

Further experimental efforts are now aimed at finding strategies to artificially manipulate sensitivity to metabolic hormones and plasticity of their target brain areas. The need of tools to harness and control the dynamic potential of the brain-metabolism interplay has a great therapeutic potential. Indeed, severe obesity is usually accompanied by loss of response to leptin and insulin.

The basic idea is based on restoring a juvenile-like status of heightened plasticity, which would help in restoring an optimal metabolic set-point, ultimately leading to body weight normalization.

## The non-cell autonomous regulation of cerebral cortex plasticity by homeoprotein Otx2 (Alain Prochiantz)

The conception that development brings individuals to a state of perfection from which they will progressively depart in the course of ageing is anchored into a vision of living organisms as thermodynamic machines obeying the second law of thermodynamics. Best illustrated by Bichat in the 19th century, this conception is also present in Schrödinger’s philosophy of life or in the work of the cyberneticists. Several extremely productive consequences have emerged from the latter views, among which the spectacular post-war ascent of molecular biology. However, the 19th century has also been at the origin of a completely opposite view theorized by Claude Bernard, who proposed that life is a permanent movement of construction and deconstruction, taking place at all levels, and that adult organisms are renewed in permanence through what he poetically named "silent embryogenesis".

The recent development of stem cell research and the discovery that we regain our own weight in cells every year has lent weight to the Bernardian view of a very plastic state for all living objects. A possible physiological justification of such plasticity is that it is easier, in many instances, to replace bad cells than to repair them, and also that new elements - if in excess - could favour somatic Darwinian selection, that is adaptation through individuation.

In the case of the nervous system, in particular the mammalian one, things are slightly more complicated because, with the exception of the two main adult neurogenic regions, the dentate gyrus zone in the hippocampus and the subventricular zone, most neurons are not renewed ( Alvarez-Buylla & Lim, 2004; Deng *et al.*, 2010; Ernst & Frisen, 2015). This is possibly because they are engaged in neural networks of high physiological value that have to be maintained throughout life. However, this opens a very important issue: how is learning, and thus adaptation to the environment, made possible not only at early post-natal stages when neural networks are built, but also in the adult when they are completed.

Our laboratory has tackled this issue by working on critical periods (CPs) in the development of the mouse cerebral cortex. Without going into detail, CPs correspond to periods of time during which the cortex can easily learn from the environment and respond by adapting its neuronal circuits to the sensory inputs it receives ( Bavelier *et al.*, 2010). For example, the competition between the two eyes for binocular vision in the visual cortex 1 (V1) takes place between post-natal day 20 (P20) and P40 in the mouse. It means that closing an eye between P20 and P40 reduces its visual acuity (amblyopia), but that this functional “disconnection” does not take place if the eye is closed before P20 (plasticity onset) or after P40 (plasticity closure) ( Maffei *et al.*, 2010).

**Figure 2.**
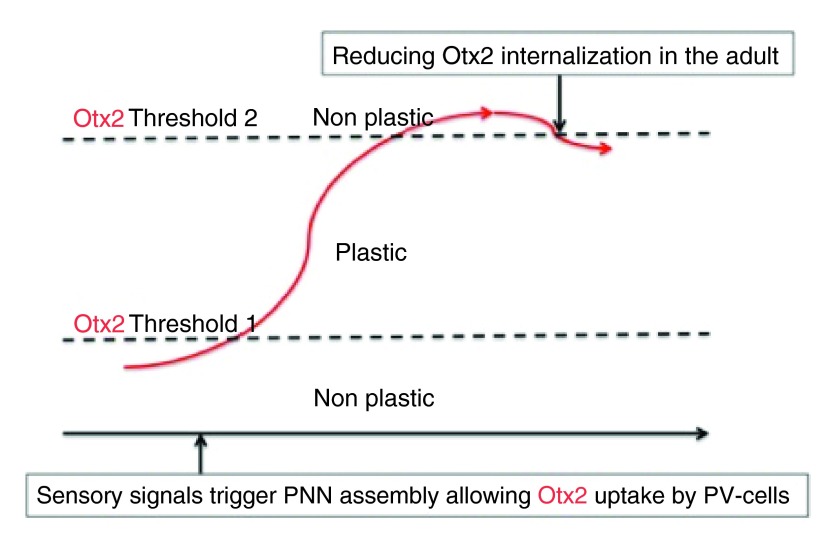
Critical period and the role of Otx2. Sensory signals, for example eye opening at P14 in the mouse, triggers Perineuronal net (PNN) assembly at the surface of Parvalbumin neurons in layers III/IV of the visual cortex. PNN complex sugars specifically recognize Otx2 en route from extra-cortical sources and enhance its internalization by Parvalbumin neurons, thus triggering the maturation of this class of interneurons. Otx2 reaches a first intracellular concentration threshold that opens plasticity and accumulates until a second threshold closes plasticity. The maintenance of an adult non-plastic state requires the continuous import of Otx2 from extra-cortical sources and blocking the latter import reopens a window of plasticity in the adult.

The passage from no plasticity (P20) to maximal plasticity (P30) and no plasticity again (P40) is due to the progressive maturation of inhibitory Fast-Spiking Parvalbulmine neurons (FSPV-cells) and to an ensuing shift in the in Excitatory/Inhibitory (E/I) balance ( Hensch & Fagiolini, 2005). We discovered, in collaboration with the group of TaKao Hensch, that the homeodomain transcription factor Otx2 is necessary and sufficient to open plasticity at P20 and close it at P40. We proposed that Otx2 progressively accumulates within FSPV-cells, opens plasticity at a first concentration threshold and closes it at a second one ( Figure 2), ( Beurdeley *et al.*, 2012; Spatazza *et al.*, 2013; Sugiyama *et al.*, 2008). Throughout adulthood, Otx2 concentration normally remains above the second threshold maintaining the system in a non-plastic state (or a poorly plastic one).

Importantly, Otx2 is not produced by FSPV-cells and is thus imported from external sources. Among the possible sources is the choroid plexus, which synthesizes Otx2 and secretes it into the cerebro-spinal fluid. It was demonstrated that indeed Otx2 is imported from this source and that its levels within FSPV-cells is reduced upon recombination of the Otx2 locus, specifically in the choroid plexus ( Spatazza *et al.*, 2013). This reduction in the adult brings Otx2 concentration back below the second threshold and reopens plasticity allowing one to restore normal binocular vision to previously amblyopic mice, or (and less nicely) to induce amblyopia in the adult.

Without elaborating further, this finding, which, in fact, might be generalized to other regions of the cerebral cortex, demonstrates that plasticity is the default state of the adult cortex and that the continuous arrival of Otx2 from the choroid plexus (and possibly other sources) into FSPV-cells maintains the cortex in a non-plastic state. It also suggests that transient local changes in Otx2 levels could reopen windows of plasticity in the adult.

In other words a tightly-controlled potential for adaptive change continues to persist in the adult nervous system of higher vertebrates, implying both the need for stability, but also the possibility for local renewal.

## The dynamic encoding and processing of morphological information during development and regeneration (Daniel Lobo)

During development and after injuries, organisms orchestrate a highly dynamic process to converge into predetermined forms and shapes – a target morphology whose specification must reside within the organism. Understanding how this target morphology is encoded, stored, and retrieved is essential to explaining the mechanisms controlling these multicellular processes. Moreover, the characteristic feedback loops and nonlinear dynamics typical of biological regulation prevent us from easily inferring the complex processes controlling these systems. Computer science theory and practice can readily assist us with the mathematical formalisms necessary to rigorously describe and analyse these processes and with the computational tools to automate the discovery of such complex dynamic regulatory networks. These computational approaches may represent an inflection point in our goal to explain the formation and repair of biological form and shape, and facilitate the much sought after biomedical and synthetic biology applications.

The regeneration of planarian worms is one of the most extraordinary examples of dynamic development and restoration of form and shape. Planarians are freshwater flatworms with a complex morphology, possessing a nervous system with a true brain, a diverse set of sensory organs including eyes, a complex musculature, and a branched digestive system. Despite their complex morphology, planarians can regenerate a complete body from almost any amputated piece, growing anew missing organs and resizing existing ones ( Lobo *et al.*, 2012). These remarkable abilities, whose principles are not yet well understood, makes the flatworm an extraordinary model system for studying the dynamics of morphological formation and repair.

**Figure 3.**
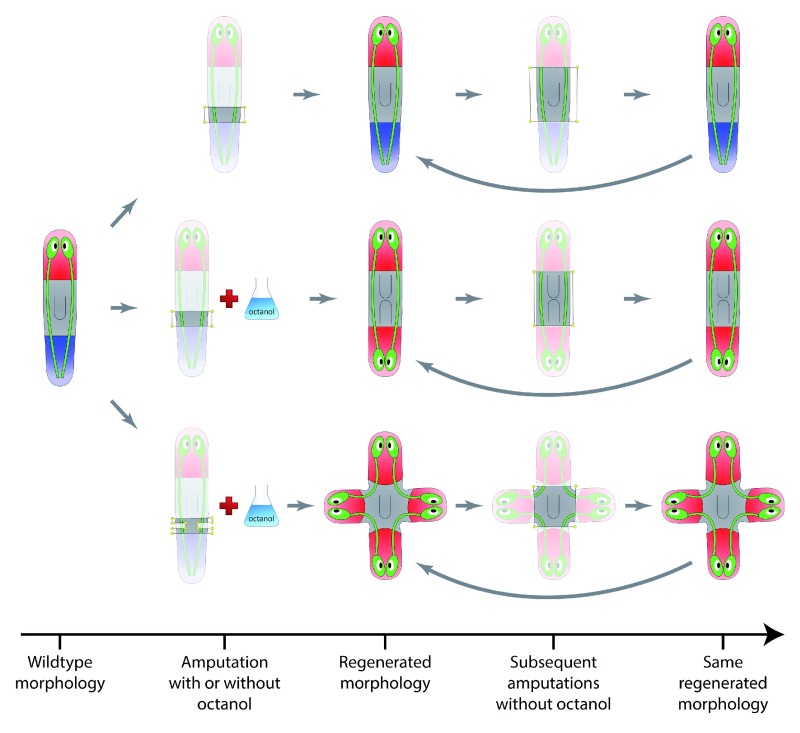
The plasticity of the target morphology in planarian worms. The regenerating phenotype from an amputated planarian trunk piece can be locally and permanently altered by non-genetic perturbations, suggesting a linear encoding of the target morphology outside the genome. Experiments extracted from Planform ( Lobo *et al.*, 2013a).

Interestingly, although the target morphology of the wild-type planaria is a head-trunk-tail phenotype, a transient blockage of gap junction communication with octanol after specific surgical amputations can cause the regeneration of permanent multi-headed morphologies ( Figure 3): subsequent amputations produce the same multi-head morphology, even without the application of any drug ( Lobo *et al.*, 2014b; Oviedo *et al.*, 2010). Remarkably, neither the octanol treatment nor the surgical manipulation directly cause any genetic change in the worm, suggesting that the target morphology to be regenerated is not encoded in the genome, but possibly by chemical morphogens, mechanical stresses, or electrical signals maintained within the worm’s body. Moreover, the ability to alter these morphological encodings with localized interventions (each surgical amputation produces a head in that location) points towards a linear encoding of the target morphology, similarly to one-to-one expression patterns, such as the homeotic gene expression patterns in the *Drosophila* embryo, which can be altered with localized interventions. In contrast, small alterations in nonlinear encodings, characteristic of gene regulatory networks such as the *Drosophila* gap gene network, produce large defects in the resultant morphology.

Moreover, the nonlinear interactions and feedback loops in nonlinear encodings represent an extraordinary barrier to understand, and possibly to control, the dynamics of development and regeneration. Reverse-engineering dynamical systems with feedback loops is indeed very hard, and no analytical solution exists to solve this inverse problem. Fortunately, novel heuristic computational methods together with the increase of available computational power are showing promising results in aiding in the discovery of such regulatory mechanisms ( Villaverde & Banga, 2013).

In order to apply computational methods to the discovery of dynamic networks from morphological experiments, the manipulations and resultant phenotypes need to be encoded in a mathematical format comprehensible for the computer, a non-trivial task for developmental and regenerative datasets. To this end, mathematical ontologies for morphological and experimental data have been proposed for planarian and limb regeneration experiments, and, based on these formalizations, centralized databases have been curated containing thousands of experiments published in the literature ( Lobo *et al.*, 2014a; Lobo *et al.*, 2013b). These freely available resources allow any scientist to instantaneously search for the resultant morphologies from specific genetic knock-downs or pharmacological interventions among thousands of experiments in the literature. More importantly, the mathematical nature of these resources makes the application of computational approaches amenable to reverse-engineer the underlying mechanisms explaining the experimental results in the datasets.

Recent advances in machine learning and artificial intelligence have facilitated the application of automated inferring methods for the automatic discovery of dynamic regulatory networks directly from the morphological outcomes of surgical, genetic, and pharmacological experiments ( Lobo & Levin, 2015; Lobikin *et al.*, 2015). These methods are based on the *in silico* evolution of dynamic networks that can recapitulate, in a virtual environment, the precise resultant phenotypes after applying the same surgical and genetic perturbations contained in the *in vivo* dataset of experiments. This approach has been successfully applied to the main head versus tail planarian regeneration experiments published in the literature, resulting in the reverse-engineering of a comprehensive dynamic regulatory network of planarian regeneration and demonstrating the power of these automated computational methods.

In summary, the emergent dynamics of development and regeneration represent a current challenge to understand these processes. To this end, it is important to differentiate between two types of morphological encodings present in biological systems: nonlinear encodings typically stored in the genome, and linear encodings (possibly originally formed during development from the nonlinear encoding) typically stored in chemical, mechanic, or bioelectric patterns. A linear encoding is easy to alter or repair and amenable for direct medical interventions. In contrast, a nonlinear encoding is hard to understand and control. For this reason, novel automatic computational tools are being proposed to aid us in the reverse engineering of these nonlinear encodings, such as the dynamic regulatory networks controlling planarian regeneration. This approach will pave the way for the understanding and control of the outstanding capacity of living organisms to self-construct and self-repair.

## The importance of variance in the nervous system (Angelika Steger)

In the digital world we have a very precise understanding of how information is stored: a single bit stores a value of either 0 or 1, and the combination of bits can be used to store more interesting facts. On the other hand, even after decades of intensive research, our understanding of how information is stored in the brain is still embarrassingly poor. Naturally, we do know basics facts: in the brain a neuron conveys information to its neighbours in form of spike trains, that is, sequences of electric pulses. Pulses *per se* seem to not differ from each other, so the information must lie either in the number or the timing of the pulses. Both options do not exclude each other, and it is non-trivial to distinguish between the two ( Stein *et al.*, 2005). There are also many indications that a single bit of information is not stored by a single neuron, but instead by a large group of neurons. This is known as population coding. Unfortunately, again, so far we have no idea of how exactly this is done.

There is no obvious way of how to resolve this riddle. Two approaches seem natural: (i) collect as many neurophysiological data as possible, and (ii) develop (mathematical) models that can explain phenomena observed in the brain. A challenging part, however, remains: bridge the gap between these approaches. A major hindrance here is that biological reality comes with a broad range of varieties: many different neuron classes and even within the same class varying properties (threshold potential, membrane capacity, etc.), synaptic connections that come with a wide range of synaptic strengths and failure rates of up to 80%, structural properties of the underlying brain tissue that are similar but not identical throughout neo cortex, and so on.

A standard approach in mathematics and many other disciplines is to first understand how a system works in a ‘pure’ setting and then generalize it step by step in order to transfer it to the noisy ‘real world‘ scenario. In brain research, however, a challenging and still not well resolved first step is: what is an appropriate ‘pure’ setting? Before we know how the brain functions it is hard to come up with classifications that differentiate between ‘meaning’ and unintended variations and imperfections that can be summarized as ‘noise’. But without such a classification it seems impossible to come up with a model that can subsequently be matched successfully to experimental observations. This circular argument nicely explains the problems we are facing. A common approach to overcome this is to start with very simple models -- and to then learn from failures.

In our work we study the effect of adding variance to neuronal processes ( Lengler *et al.*, 2013). Similarly, as was observed in other contexts ( Wiesenfeld & Moss, 1995) we show that variance within defining properties of neurons and their synapses is not a handicap of neural systems but that, instead, predictable and reliable functional behaviour of neural systems depends crucially on this variability. In particular, we show that higher variance allows a recurrently connected neural population to react more sensitively to incoming signals, and processes them faster and more energy-efficiently. This challenges a widely spread assumption that variability of neurons in the brain is a defect that has to be overcome by synaptic plasticity in the process of learning. Instead, it might well be that the variability is actually a feature and not a bug!

Another and perhaps even more surprising result is that variability in the neuronal parameters guarantees stability. We illustrate this at one of the basic processes that occur in neural processing: an external input activates some brain area and this activation spreads due to local recurrent connections. While an initial spread of activity may well be a desired feature, it certainly needs to be stopped before an epileptic seizure occurs. As it turns out, it is not at all easy (neither in simulations nor in theory) to come up with models that exhibit such behaviour, as either inhibition kicks in too early (and in turn nips all activity in the bud) or inhibition is too weak to ever have a sizeable effect and systems thus do not have a decorrelating effect as observed in nature ( Ranganathan & Koester, 2011). We could overcome this well-known phenomenon by adding a lot of variance to the system: variance in the definition of the neuron parameters as well as variance in the reliability of the synaptic connections. In fact, our simulations show that an increased level of variance actually leads to more and not less stability in the dynamics of the considered network, and that it is most helpful to have variance in many parameters and not just one. It remains a challenging question to understand whether neuronal plasticity actually enhances variance purposefully in order to strengthen these effects.

## General discussion

### How to conceive of life-long development?

The format of this conference enabled the juxtaposition and comparison of different model systems, which are being used to uncover the physiological underpinnings of a dynamic architecture of life. One conclusion that can be drawn is that very different balances between change on one side and constancy on the other have evolved in animals and plants: On one end of the spectrum, one might locate animals, showing a capacity for life-long regeneration of their body or appendages. On the other end of the spectrum animals and plants can be found, which possess a much more limited regenerative faculty, but harbour a potential for life-long individual development through adaptive change.

The reader might comment that this distribution has been known and has been well accepted for most of the 20 ^th^ century. However, the increasing body of evidence provided for the continuous capacity for plasticity in higher order species puts novel demands to understand the dynamics inherent in living systems, whose high degree of structural and functional complexity has been understood to exclude most change in the mature organism. How is the level of control achieved that permits adaptive change, but bars excessive alterations that would threaten the functionality of complex organs, such as the mammalian nervous system? How are the adaptive responses organized? Do they follow a pre-established hierarchy or is their sequence a reflection of the particular demands to the organism? The latter implies that changes can be elicited at any functional level, for example genes, proteins, cells, tissues or, in the case of the brain, also different parts of the organ, and are then transmitted to the other levels to achieve a coordinated adaptation (for discussion see Morange, 2009). This is also relevant in the context of therapeutic applications addressed below.

### How does individual adaptation translate?

Epigenetics seems to provide an important link transforming individual adaptations garnered during the existence of the organism into a property that might be selected for and thus contribute to advances at the species level. The extensive knowledge of epigenetics in plants makes them particularly attractive to discuss these questions. Interestingly, the caution that still prevails in the plant field in accrediting epigenetic change a role in evolution seems to be contrasted by an overly eager reception of evidence for epigenetic changes in animals, which does not stand up to the application of more rigorous criteria. As Ueli Grossniklaus discusses, the establishment of unambiguous parameters for the intergenerational passage of epigenetic modifications is an important pre-requisite for clarifying the importance of epigenetics in animal studies. This example also demonstrates that direct encounters like this conference might be helpful in getting essential know-how across disciplinary divides.

Marie Mirouze states in her contribution that epigenetically induced genetic variation (e.g. the activation of transposons) amongst cellular constituents of an organism can be advantageous and underscores the importance of epigenetic inheritance in rendering differentiation transferable from mother to daughter cells. As Alain Prochiantz discusses, variability amongst newly generated cells might also convey a selective advantage to specifically adapted individual cells within the nervous system. Interestingly, the efforts to model the enormous complexity of information storage in the brain lead Angelika Steger to conclude that variance of neuronal parameters is not something to be necessarily eliminated, but rather might be favourable for improving the overall efficiency of signalling. This provokes the question to which degree variance amongst cellular constituents of organisms, regardless of its origins, is productive and desired and how it might be controlled and selected for.

What do we make of the fact that the regenerative capacity is much more limited in mammals? Are the claims made about the application of the insights gained about regeneration in different animal models for therapeutic interventions in humans very ambitious or justified in view of the fact that genetic pathways underlying regenerative processes are widely conserved, as Brigitte Galliot emphasizes? Do therapeutic measures have to address entire physiological processes or might it be sufficient to tweak one or a few particular parameters, implying a plastic capacity of the system that can be induced in different ways to respond in a therapeutically desirable fashion?

### The role of the environment

In simple as well as complex organisms, adaptive change and an environment guiding and shaping that change seem to go hand in hand. While in the case of *Hydra* and plants modelling the environment in an ecologically relevant fashion is relatively easily to achieve, it is more demanding to do so in the case of rodents. In the neurosciences the recourse to a so-called “enriched environment” has become the method of choice to provide an experimental environment that is demanding to the animals on different levels. As the work by Marco Mainardi and others shows, specific changes are elicited in the nervous system in response to a range of physiological stimuli. One might wonder which changes might become possible if the complexity of the environment was raised beyond that of an enriched environment to a more natural environment and in which ways this might promote differences in individual animals (e.g. Freund *et al.*, 2013).

### Same terms, different contexts?

As the contributions to this review show, the use of central terms - epigenetics, plasticity, and regeneration - is partially overlapping, as different authors emphasize different aspects of the processes of adaptive change they are studying. In reflection of a variable use of concepts in biology, in the history and philosophy of science, the argument has been put forward that it is the differential applicability of biological concepts, which renders them useful in propelling forward a largely empirical science like biology, which advances by posing ever more refined questions about the character and content of its concepts (see the analysis of the concept of the gene during the twentieth century by Rheinberger *et al.*, 2015). However, the variable and widespread use of biological concepts has also been criticized, as implying a loss in precision, ultimately rendering them too vague for proper scientific exchange. In the case of plasticity, suggestions have been made to specify the use of the concept in the neurosciences in order to provide an appropriate framework for further empirical studies (e.g. Will *et al.*, 2008). In view of the fact that the knowledge of the inherent dynamics of biological systems is steadily increasing, it might be necessary to develop a novel terminology, which takes into consideration the recent advances and introduces unambiguous criteria for the use of specific concepts in different fields. Modelling approaches like the one presented by Daniel Lobo could contribute to a discussion on universally applicable criteria, which are sufficiently stringent to guide researchers in the common use of defined terms.
