# Supplementary material for: A dynamic architecture of life
Source: F1000Res. 2015 Nov 18;4:1288. [Version 1] doi: 10.12688/f1000research.7315.1 (PMC4760269; doi:10.12688/f1000research.7315.1)
Supplement: Peer review history

# Peer review: Referee response for version 1

**DOI:** 10.5256/f1000research.7883.r12521

**Published:** 2016-02-18

**Authors:** Pierre J Magistretti

**Affiliations:** 1 Brain Mind Institute, Swiss Federal Institute of Technology in Lausanne (EPFL), Lausanne, Switzerland

A group of experts met at Accademia dei Lincei to discuss new concepts emerging from experimental data on the dynamic process of living organisms particularly as they relate to differentiation, adaptation, plasticity and memory. The report provides snapshots from selected fields of biology illustrating the paradigm shift that has occurred over the last two decades concerning the dynamics of a variety of molecular and cellular processes. The view that emerges is one of permanent instability that allows for dynamic adaptations to environmental cues. The set of process underlying this instability most likely provides the key to species survival and adaptation, but also, and just as importantly, the emergence of individuality. The paragraphs reporting on the various presentations are sharp and informative and the introductory and concluding ones provide a faithful synthesis of the questions at stake and their elaboration. The only aspect that could have been treated more directly would have been that of synaptic plasticity in the adult which represents undoubtedly an important element of the dynamic architecture of life. 

This review article represents a remarkable scholarly effort to provide novel insights on a paradigm shift about our understanding of the dynamics of life.

I have read this submission. I believe that I have an appropriate level of expertise to confirm that it is of an acceptable scientific standard.

# Peer review: Referee response for version 1

**DOI:** 10.5256/f1000research.7883.r11799

**Published:** 2016-01-29

**Authors:** Richard Festenstein, Petra Hajkova

**Affiliations:** 1 Medical Research Council Clinical Sciences Centre (MRC CSC), Faculty of Medicine, Imperial College London, London, UK; 2 Division of Brain Sciences, Faculty of Medicine, Imperial College London, London, UK

This report summarises the key points of a recent meeting bringing together scientists working on diverse biological systems linked mainly by their approach to understanding the dynamic aspects of biological states. The report highlights the importance of reversibility of differentiation states and cellular responses to external stimuli as well as the importance of this in the context of adaptive evolution. Clearly there are many unanswered questions and the historian’s perspective pointed out that there is much more experimental work required to formulate general principles linking the described systems. Meetings such as these are of great value in allow abstraction of such principles and are to be encouraged. The inclusion of an historian in such meetings is an innovative approach which could be developed further to allow a real in-depth analysis of how ideas evolve over time.

We have read this submission. We believe that we have an appropriate level of expertise to confirm that it is of an acceptable scientific standard.
